# Noninvasive prenatal diagnosis of duchenne muscular dystrophy in five Chinese families based on relative mutation dosage approach

**DOI:** 10.1186/s12920-021-01128-1

**Published:** 2021-11-22

**Authors:** Ganye Zhao, Xiaofeng Wang, Lina Liu, Peng Dai, Xiangdong Kong

**Affiliations:** 1 Genetics and Prenatal Diagnosis Center, The Department of Obstetrics and Gynecology, The First Affiliated Hospital of Zhengzhou University, Zhengzhou, 450052 Henan China; 2 Hunan Research Center for Big Data Application in Genomics Genetalks Inc., Changsha, 410152 Hunan China

**Keywords:** Noninvasive prenatal diagnosis, Duchenne muscular dystrophy, Relative mutation dosage, Cell free DNA, cfBEST

## Abstract

**Background:**

Relative haplotype dosage (RHDO) approach has been applied in noninvasive prenatal diagnosis (NIPD) of Duchenne muscular dystrophy (DMD). However, the RHDO procedure is relatively complicated and the parental haplotypes need to be constructed. Furthermore, it is not suitable for the diagnosis of de novo mutations or mosaicism in germ cells. Here, we investigated NIPD of DMD using a relative mutation dosage (RMD)-based approach—cell-free DNA Barcode-Enabled Single-Molecule Test (cfBEST), which has not previously been applied in the diagnosis of exon deletion.

**Methods:**

Five DMD families caused by *DMD* gene point mutations or exon deletion were recruited for this study. After the breakpoints of exon deletion were precisely mapped with multiple PCR, the genotypes of the fetuses from the five DMD families were inferred using cfBEST, and were further validated by invasive prenatal diagnosis.

**Results:**

The cfBEST results of the five families indicated that one fetus was female and did not carry the familial molecular alteration, three fetuses were carriers and one was male without the familial mutation. The invasive prenatal diagnosis results were consistent with those of the cfBEST procedure.

**Conclusion:**

This is the first report of NIPD of DMD using the RMD-based approach. We extended the application of cfBEST from point mutation to exon deletion mutation. The results showed that cfBEST would be suitable for NIPD of DMD caused by different kinds of mutation types.

**Supplementary Information:**

The online version contains supplementary material available at 10.1186/s12920-021-01128-1.

## Background

Duchenne/Becker muscular dystrophy (DMD/BMD) are X-linked recessive diseases that are caused by defects in the *DMD* gene [1]. It is one of the most common severe, untreatable neuromuscular disease, that presents as a progressive muscle disorder. DMD is a lethal inherited muscle disease mostly in young boys, with an incidence at birth of 1/3800–1/6300 [2]. BMD is a milder form with onset later in childhood and a low incidence of 1 in 20,000–30,000 males [3]. As no curative therapy is currently available, the importance of prevention is paramount. Traditional invasive procedures including chorion villus sampling and amniocentesis could raise anxiety in pregnant women and carry the risk of miscarriage or injury to the fetuses and the mothers [4]. Noninvasive procedures would be more easily accepted by pregnant women and their families [5].

The discovery of cell free fetal DNA (cffDNA) in the maternal plasma and the next generation sequencing (NGS) technology made the non-invasive prenatal testing (NIPT) possible [6, 7]. NIPT is already widely used in screening for aneuploidies [8–11]. Entire fetal genomes are represented in the maternal plasma at a constant relative proportion, which makes it possible to diagnose fetal genetic disorders prenatally in a noninvasive way [12]. To date, different noninvasive prenatal diagnosis strategies have been presented including the relative mutation dosage (RMD) approach and the relative haplotype dosage analysis (RHDO) [13–16]. The RHDO approach has been successfully used in DMD [17–21], but the procedures are relatively complicated [17, 22].

A new technology called cell-free DNA barcode-enabled single-molecule test (cfBEST) is able to detect monogenetic disease noninvasively with a high sensitivity and specificity [19]. With the strategy of cfBEST, it is possible to count the original and real molecules accurately by reducing the influence of random sequencing errors, unbalanced PCR amplification, and different PCR amplification efficiency between fetal and maternal cell free DNA (cfDNA). Here, we report on the RMD-based NIPD for DMD caused by point mutations or small insertions/deletions by target-position sequencing of the maternal plasma using cfBEST. Moreover, after precisely mapping the deletion breakpoints of a sample with one exon deletion, we also inferred the genotype of the fetus using cfBEST. All of the results were confirmed with subsequent invasive procedures.

## Materials and methods

### Study design

**Fig. 1:**
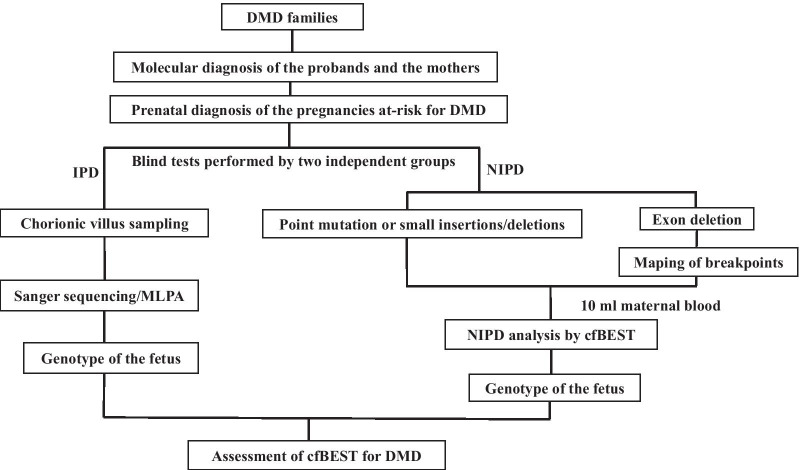
The workflow of this study. First, we collected samples from DMD families and identified the mutation genotypes of each case. Then, noninvasive prenatal diagnosis and invasive prenatal diagnosis were blindly conducted by two independent groups. Finally, we assessed the performance of cfBEST for DMD

The study workflow is illustrated in Fig. 1. This clinical research study was conducted at the Prenatal Diagnosis Center of the First Affiliated Hospital of Zhengzhou University. DMD families that had a previous child affected with DMD from their first pregnancy were recruited to the study. After genetic counselling, all the probands and the mothers elected to undergo molecular diagnosis to identify the causative pathogenic *DMD* mutations. For clinical management of second pregnancies, NIPDs were blindly performed in parallel with invasive prenatal diagnosis (IPD). NIPD was performed through cfBEST after mapping the breakpoints. IPD was performed through chorionic villus sampling (CVS) followed by Sanger sequencing or Multiplex-ligation dependent probe amplification (MLPA). Finally, we assessed cfBEST for DMD through comparing the results of NIPD with IPD.

### Sample collection and DNA extraction

All subjects gave their informed consent for inclusion before they participated in the study. Samples were collected with consent from subjects at the First Affiliated Hospital of Zhengzhou University. A cohort of five DMD families were recruited. The characteristics of the families are summarized in Table 1. A healthy man with no *DMD* mutation was chosen to be the control. Each family cohort consisted of a proband and his mother with a second new singleton pregnancy. Blood samples (2 mL) from the probands and the control were collected. In addition, 10 mL blood samples were collected from pregnant women and 5 mg of chorionic villus from the fetuses were collected via CVS. DNA in white blood cells of the proband and the normal healthy control sample were extracted using the DNeasy Blood Tissue Kit (Qiagen, Dusseldorf, Germany). Genomic DNA was extracted from fetal cells using the Lab-Aid Nucleic Acid (DNA) Isolation Kit (Zeesan, Xiamen, China) according to the manufacturer’s instructions. Blood samples (10 mL) from the pregnant women were centrifuged twice to collect the plasma, which was used to extract cfDNA using the QIAamp Circulating Nucleic Acid Kit (Qiagen, Dusseldorf, Germany).

**Table 1:** Summary of the information of the five DMD families

Family ID	Mutation (hg19, NM_004006)	Maternal genotype	Gestational age (week)	Maternal age (year)
D1	c.5697_5698insA (p.Lys1899LysfsTer7)	het^a^	12	31
D2	c.1231A > T (p.Lys411Ter)	het	12	29
D3	c.1929G > A (p.Trp643Ter)	het	12	26
D4	c.2305G > T (p.Glu769Ter)	Normal	12	31
D5	Exon 12 deletion	het	11	32

^a^het: Heterozygous

### Mapping breakpoints of the proband with exon 12 deletion of the DMD gene

**Fig. 2:**
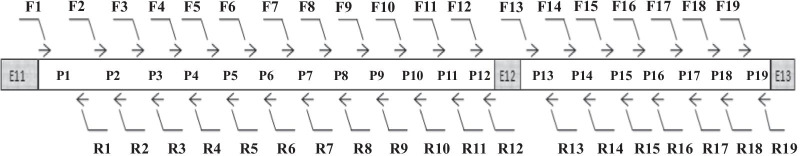
Localization of the 19 parts (P1-P19) between exon 11 and exon 13 of the *DMD* gene divided by the relevant primers. F: forward primer, R: reverse primer. E11-E13: exon 11-exon 13 of *DMD* gene

We designed 19 pairs of primers (Additional file 1: Table S1) between exon 11 and exon 13 of the *DMD* gene (GenBank NG_012232) at contiguous intervals of approximately 1.25 kb (Fig. 2). This enabled division of the region between exon 11 and exon 13 into 19 parts (P1-P19) (Fig. 2), with each part being amplified by its relevant primers. PCR was conducted using the primers designed above in the proband and the healthy normal control sample. The presence/absence of these amplified products were verified by agarose gel electrophoresis. If the parts could not be amplified for the proband, the primers would be in the deletion region, as the primers could not bind to the templates. It was then possible to narrow down the region that included the breakpoints. Finally, genomic regions spanning the breakpoints were amplified and sequenced using the new primers (Additional file 1: Table S2) designed according to the results of the above agarose gel electrophoresis.

### Primers design for the DMD cfBEST assay

Four primers were designed for each DMD mutation site based on the principle of cfBEST [19] (Additional file 1: Table S3). Furthermore, cfBEST primers for chromosome Y had been designed to detect fetal gender (Additional file 1: Table S4).

### DNA sequencing library preparation and sequencing

cfBEST tag adaptors were ligated to cfDNA after A-tailing was added to cfDNA using the KAPA Hyper Prep Kit (Kapa Biosystems, Boston, USA). The pre-library amplified from cfDNA with an index primer and a universal primer was split into two parts [named “F” and “R”, Additional file 2: Fig. S1 shows only the “F” part)] to be amplified separately with two rounds of PCR. The two PCR products were pooled to execute a third PCR with universal primers U1 and U2 (Additional file 2: Fig. S1). Finally, the sequencing libraries from the three rounds of PCR were subjected to massively parallel sequencing on the NextSeq CN500 (Illumina, San Diego, USA).

### Bioinformatics analysis and genotyping by cfBEST

The noise sequences were eliminated using the bioinformatic filtering step. After preprocessing of the raw sequences with barcode trimming, adapter trimming, and primer recognition, the preprocessed reads were aligned to hg19 and further filtered. After calling the consensus sequence, the allele would be counted. The allele count was modeled in a mixed binomial distribution, and the fetal cfDNA fraction was deduced using an EM algorithm with the 109 SNP [19]. Fetal gender was determined via the results of allele count on the Y chromosome, as there would be almost no count for female fetuses. The expected ratio of mutant alleles in plasma would be deduced according to the condition of the pregnant woman and the fetus (Additional file 1: Table S5). The final genotyping results would be deduced using an in-house R-script.

### Invasive prenatal diagnosis

Fetal cells were retrieved by CVS at around 12 gestational weeks. The mutations were tested by PCR and Sanger sequencing or MLPA using standard protocols in the laboratory. Maternal contamination was excluded via quantitative fluorescent polymerase chain reaction (QF-PCR) using the GoldeneyeTM DNA ID System 20A Kit (Peoplespot, Beijing, China) in accordance with the manufacturer’s instructions.

## Results

### Family cases

For Family D1, the causative mutation was identified as insertion A between physical position 32,361,292–32,361,293. The causative mutations were nonsense mutations for Families D2-D4. Family D5 had an exon 12 deletion. All of the pregnant women from the DMD families except Family D4 were *DMD* carriers with a gestational age of about 12 weeks. For Family D4, the mutation of the proband may have been de novo or the mother of the proband may have had germline mosaicism. The age of the pregnant women ranged from 26 to 32 years of age. More details are shown in Table 1.

### Map of the breakpoints

**Fig. 3:**
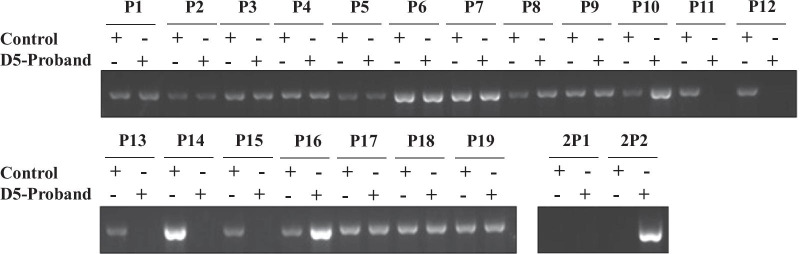
The results of PCR using the same primers with different templates (normal control, and proband of Family D5). P11-P15 had no signal in proband of Family D5 compared with the normal control whose *DMD* gene was totally normal. 2P1 had no signal in both the control and proband of family D5. 2P2 had no signal in the normal control, but there was signal for proband of Family D5. All of these indicate that the breakpoints were localized in part 2P2

The specific primers in Additional file 1: Table S1 were used to amplify the genomic DNA of the proband from Family D5 and a normal control sample, respectively. The results showed that there was no signal in parts P11–P15 of the proband of Family D5, which indicated that the breakpoints of this sample may be localized in these parts (Fig. 3). In order to accurately locate the breakpoints, two more reverse primers were designed in the primers R14–F16 region (Additional file 1: Table S2). The forward primer of P11 was paired with the two new reverse primers respectively to amplify the genomic DNA (gDNA) of the proband from Family D5 and the control (Fig. 3). There was no signal in either 2P1 or 2P2 in the control sample, with the PCR products being about 10 kb and too long to be amplified, as the PCR conditions were suitable for PCR products with about of around 1.25 kb in size. There was no signal in the region 2P1 in the proband from Family D5, which indicated that this region was deleted. The signal of 2P2 in the proband from Family D5 indicated that the breakpoints were localized in this region. Later, the PCR products of the part 2P2 of the proband from Family D5 was sequenced and aligned with the *DMD* gene to map the breakpoints. Finally, the breakpoints of this sample were localized at 32,625,751 and 32,635,984 on chromosome X (g.32625752_32635983del) (Table 2).

**Table 2:** The results of the five DMD families undergoing NIPD by cfBEST and invasive prenatal diagnosis

Family ID	Physical position (X chromosome, hg19)	DNA input (ng)	Mutation unique reads	Total unique reads	Mutation ratio (%)	Fetal DNA fraction (%)	Fetal gender	Fetal genotype	
								cfBEST	IPD^b^
D1	32,361,292–32,361,293	17.97	154	380	40.53	13.06	Female	N/N^a^	N/N
D2	32,662,349	28.10	1539	2941	52.30	11.70	Female	N/M^c^	N/M
D3	32,583,882	18.38	1062	2089	50.80	14.70	Female	N/M	N/M
D4	32,519,947	26.48	0	3476	0.00	3.00	Male	N	N
D5	32,625,751, 32,635,984^d^	34.78	346	695	49.78	10.20	Female	N/M	N/M

^a^N normal wild-type allele

^b^IPD invasive prenatal diagnosis

^c^M mutant allele

^d^The two breakpoints of sample D5

### NIPD of DMD

NIPD was performed on plasma samples from the five second pregnancies at risk for DMD using the cfBEST assay targeting the five different *DMD* mutations. Plasma samples were sent to an independent laboratory for analysis without information on the fetal genotypes determined by IPD. The fetal fraction of the plasma tested was > 5%, except for the sample from Family D4, which was 3.00%. Finally, the NIPD results showed that the fetus was female and did not inherit the maternal mutation for Family D1. For families D2, D3 and D5, the fetuses were females and carried the same mutations as the proband. For family D4, the fetus was male and did not inherit the maternal mutation.

### Invasive prenatal diagnosis through CVS

**Fig. 4:**
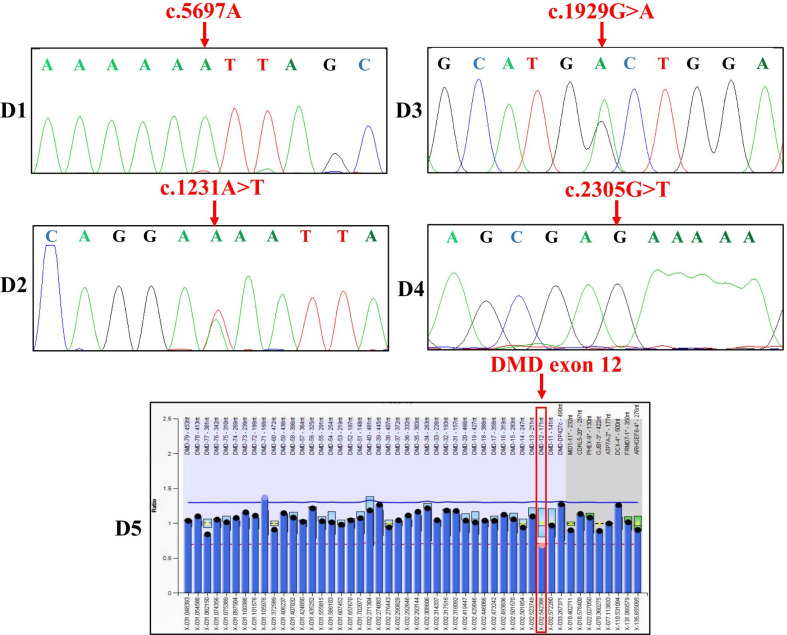
The invasive prenatal diagnosis results of Families D1-D5 with Sanger sequencing (Families D1-D4) or MLPA (Family D5). For Families D1 and D4, the fetuses did not inherit the maternal mutation. For Families D2 and D3, the fetuses were female carriers. The fetus was a carrier with exon 12 heterozygous deletion for Family D5

For Families D1-D4, genotyping of the fetuses took place via Sanger sequencing (Fig. 4), while, MLPA was used to genotype the fetus for Family D5 (Fig. 4). The IPD results were consistent with the deduced fetal genotypes determined for each of the five pregnancies by NIPD (Table 2).

## Discussion

In the current study, both NIPD and IPD were performed on five pregnant women from DMD families caused by different mutation types to assess the performance of the cfBEST assay for quantification of different kinds of *DMD* mutation types in the cfDNA of pregnancy plasma. In all five cases with three different mutation types, including substitution mutation, small insertion, and exon deletion, the fetal genotypes were correctly inferred by the mutation percentage according to the cfBEST assay. The fetal genotypes determined by cfBEST were consistent with the IPD results. The results showed that cfBEST was suitable for different kinds of mutation types.

DMD is the second monogenic disease, after β-Thalassemia, that has been noninvasively detected using cfBEST [19]. This was the first application of cfBEST for exon deletion and X-linked diseases. Our study further indicated that cfBEST can be a universally applicable system for any other monogenic diseases with minor modifications.

Expanded noninvasive prenatal testing has already been used to screen chromosome aneuploidy and genome copy number variations regarding several mega base pairs [9]. Point mutations and small indels can be detected through the strategy of RHDO and RMD [23]. However, there are few effective methods to detect the copy number variation (CNV) with a size of around several dozens base to mega base using the RMD based strategy. We localized the breakpoints in the *DMD* gene using the proband’s genomic DNA, then cfBEST was used for noninvasive detection. The information of the breakpoints could be used for conventional prenatal diagnosis and preimplantation genetic diagnosis (PGD), providing a double guarantee for conventional methods. As reported previously [19], a minimum of 1000 unique reads were required for precisely calculating mutation ratios. In our study using the cfBEST approach, the total unique reads of Families D1 and D5 were 380 and 695, respectively, which were lower than the minimal unique reads. However, the fetal genotypes deduced by cfBEST were in accordance with the IPD results (Table 2). The high concentrations of the cfDNA from these two cases, 13.06% and 10.2% respectively, compensated the requirement of > 1000 total unique reads, enabling accurate diagnostic results from cfBEST. This indicated that the impact of the least unique reads on the accuracy of cfBEST also relied on the fetal fraction. The threshold of the parameters could be set according to the large dataset with comprehensive consideration of all of the influencing factors in the future.

With advances in molecular genetics, mosaicism in germ cells has been recognized as a relatively frequent cause of genetic disorders. Germline mosaicism has been found in 10% of the cases in a large series of sporadic patients with DMD [24]. Mothers with no detectable mutation in their lymphocytes may still have an elevated recurrence risk due to germline mosaicism [25]. In Family D4, the pregnant woman did not carry the mutation in her lymphocytes, indicating that the proband could be a de novo mutation or the woman could have germinal mosaicism, making it essential to perform a prenatal diagnosis in this case. The fetal DNA fraction of the pregnant woman from Family D4 was 3.00%, which was lower than the cutoff of 5% set previously [19]. However, the fetus was male, according to the Y chromosome signal from the sequencing results. If the fetus had been a patient, the mutation ratio would theoretically be at least 1.50%, which could be precisely detected via cfBEST, as this test shows excellent performance in detecting samples containing 0–0.5% mutations [19]. The mutation ratio of this case was 0.00% (Table 2), which indicated that the fetus was male and did not inherit the maternal mutation. Finally, the IPD result confirmed the fetal genotype given by cfBEST. Therefore, a low fetal fraction would not impact the accuracy of cfBEST results in such cases. The minimum fetal DNA fraction required in these cases should be determined and validated in extended DMD families.

Compared with these haplotype-based strategies used to detect DMD noninvasively [20, 26], cfBEST did not require construction of the haplotypes of the parents with no need for blood collected from the father, meaning that it would be less expensive and easy to perform [17, 27]. cfBEST could also be suitable for de novo mutations or mosaicism in germ cells [27], which are invalid for RHDO. Besides, if recombination events occur, the possibility of an incorrect fetal genotype classification for RHDO would increase [17, 20]. As for large segment deletion or duplication, we could map the breakpoints and conduct the detection targeting the breakpoints just as with the sample from Family D5 using cfBEST.

This is a preliminary study and additional improvements are necessary to ensure the highest clinical sensitivity and specificity of our NIPD strategy for DMD, despite the high performance of the current assay. Firstly, as this study only involved five DMD families, more cases should be recruited to further evaluate and optimize our assay in the future. Secondly, the minimum fetal fraction and unique reads required for our analyses will be determined according to the more data acquired in the future.Besides, enrichment of the fetal DNA would improve the fetal fraction to extend the application of our method to earlier gestational ages or to cases with a low fetal fraction due to other reasons.

## Conclusion

In summary, our study demonstrated the feasibility of cfBEST for DMD caused by different kinds of mutation types including exon deletion. With further validation and innovation, our assay could become a better clinical application for noninvasive prenatal diagnosis of pregnancies at risk for DMD. Our study will provide a new approach for safely and non-invasively examining fetuses at a high risk of DMD.

## Supplementary Information

**Additional file 1**. **Table S1**. Primer sequences to map the breakpoints of the proband of Family D5; **Table S2**. Primer sequences to amplify parts 2P1 and 2P2; **Table S3**. Primers used for DMD in cfBEST. **Table S4**. Primers used for Y chromosome in cfBEST. **Table S5**. The expected mutant ratio of the plasma of the pregnant woman.

**Additional file 2**. **Fig. S1**. Schematic representation of the cfBEST method. Red dot: the site of interest; UMI: Unique Molecular identifiers; Index: sample index; Primer F1: Target-specific primer in 1st PCR; Primer F2: Target-specific primer in 2nd PCR, which is close to the site of interest; Primer U1: a universal primer of P7; Primer U2: a universal tail part of P5.

**Additional file 3**. **Fig. S2**. Full-length gels of Fig. 3 are presented in Additional file 3: Fig. S2.

## Data Availability

As public access was not consented for by the subjects in our study, the raw datasets are not publicly available. However, they are available from the corresponding author upon reasonable request.

## Abbreviations

DMD: Duchenne muscular dystrophy
NIPD: Noninvasive prenatal diagnosis
RMD: Relative mutation dosage
cfBEST: Cell-free DNA Barcode-Enabled Single-Molecule Test
BMD: Becker muscular dystrophy
cffDNA: Cell free fetal DNA
NGS: Next generation sequencing
NIPT: Non-invasive prenatal testing
RHDO: Relative haplotype dosage analysis
cfDNA: Cell free DNA
MLPA: Multiplex ligation-dependent probe amplification
CVS: Chorion villus sampling
Het: Heterozygous
N: Normal wild-type allele
IPD: Invasive prenatal diagnosis
