# Supplementary material for: Noninvasive prenatal diagnosis of duchenne muscular dystrophy in five Chinese families based on relative mutation dosage approach
Source: BMC Med Genomics. 2021 Nov 22;14:275. doi: 10.1186/s12920-021-01128-1 (PMC8607717; doi:10.1186/s12920-021-01128-1)
Supplement: Supplementary file 1 — Additional file 1. Table S1. Primer sequences to map the breakpoints of the proband of Family D5; Table S2. Primer sequences to amplify parts 2P1 and 2P2; Table S3. Primers used for DMD in cfBEST. Table S4. Primers used for Y chromosome in cfBEST. Table S5. The expected mutant ratio of the plasma of the pregnant woman. [file 12920_2021_1128_MOESM1_ESM.docx]

Table S1. Primer sequences to map the breakpoints of the proband of Family D5

| **Part** | **Primer** | **Forward primer sequence** | **Primer** | **Reverse primer sequence** |
| --- | --- | --- | --- | --- |
| P1 | F1 | GGCCTTTTGAAAGAATAAATAAGGTACATG | R1 | ACCATGATAATGGAAACTGAACAGG |
| P2 | F2 | ACTGGAGGAAGAGAGGACAGTTATTAGG | R2 | AAACTCATTTGTGACTGGAGATGTG |
| P3 | F3 | TACCCCATGGGTGATTAAGCAG | R3 | ACAGGGAGACTCTGTCTCAAAAAAA |
| P4 | F4 | GGCCACTTATTGAACTTTGATAAACG | R4 | TCACCCAATACCCACAGCAAGT |
| P5 | F5 | GAACTTGAGAGTGATGATCTCGTGTG | R5 | TCTTCTACCTATGAGCCCGTAAAAT |
| P6 | F6 | CAAATGGCTGATTTGAGGATGCTC | R6 | CCATGTTATCATTGGTACGTTAAGG |
| P7 | F7 | GTTACCAGAGGATGGGAATGCTAG | R7 | CGGCTTTTATTCAAACTATCACAGA |
| P8 | F8 | ATGCCTTTATTAGTTAGTGCCTAGCATATG | R8 | GTTGTAAGAACTTGGAACTTTGGCA |
| P9 | F9 | ACCCAGAAAACTCTGGCCTATAATTC | R9 | ATTCTACAAACACAACTCACCCAAA |
| P10 | F10 | GGTATTTTGTCCATTCCTAACAAATATGAG | R10 | TTGAAAAATGTAGAGAGAGAGGACG |
| P11 | F11 | GTGCTGCCACGAACATATGTGT | R11 | AAACTAGAAGTTGAGATGACCGCTG |
| P12 | F12 | AATTTTTCGTGAATCTAAAACAGCCA | R12 | GCCTATTATGACCTCTTTGAAAGCA |
| P13 | F13 | GGGTTGCCACATTTAGCAGACA | R13 | GGAGGGTGAACTCGTAAGGCAT |
| P14 | F14 | TTGTGTAGATAGACATTAAATCCTTGTGCC | R14 | TGAGGGTTTGTTCAGTGATATGGAA |
| P15 | F15 | CCAAGGGTATATTTGCACAGTAATGC | R15 | ACAAAAGATTATAGGCTGGTAGGGG |
| P16 | F16 | TAATGGGGTTATCTATTTTTCTTCTTGTTG | R16 | CCCAAGAACTACAACAAGACAAGGA |
| P17 | F17 | TTCCTTGATTTCATTGTCCAAAGAT | R17 | GCAGGAAGAGAATGTGATTTTTGAA |
| P18 | F18 | CTCGTTAGTCTTTCCTTATGTGTGC | R18 | AACGGAGATTTATATTGGATTTGCT |
| P19 | F19 | ATAAGCTCTGGAATGTCAAAGGCAT | R19 | GTCTAATTGTATACCATTCTCGCCC |

Table S2. Primer sequences to amplify parts 2P1 and 2P2

| **Part** | **Primer** | **Forward primer sequence** | **Primer** | **Reverse primer sequence** |
| --- | --- | --- | --- | --- |
| 2P1 | F11 | GTGCTGCCACGAACATATGTGT | 2R1 | ATACCCTTGGAATATGATGCTTGTTA |
| 2P2 | F11 | GTGCTGCCACGAACATATGTGT | 2R2 | AGTGTTCGCTATTCAGATTATGGAT |

Table S3. Primers used for DMD in cfBEST.

| **Mutation site** | | **Primer** | **Sequence** |
| --- | --- | --- | --- |
| c.5697_5698insA | | F1 | GGTATCAGTACAAGAGGCAGGCTGATGATCTC |
|  |  | F2 | AGATGTGTATAAGAGACAGGCAGGCTGATGATCTCCTGAAATGCTTG |
|  |  | R1 | TCTGGTATTGACATTCTAAAACAACATTACCTTT |
|  |  | R2 | AGATGTGTATAAGAGACAGAAACAACATTACCTTTATTTTCCTTTCATCTCTG |
| c.1231A>T | | F1 | CCATCAGGGCCGGGTTGGTAATATTC |
|  |  | F2 | AGATGTGTATAAGAGACAGCGGGTTGGTAATATTCTACAATTGGGAAGTAAGC |
|  |  | R1 | CCCATCTTGAATTTAGGAGATTCATCTGCTCTTG |
|  |  | R2 | AGATGTGTATAAGAGACAGGGAGATTCATCTGCTCTTGTACTTCAGTTTCTTC |
| c.1929G>A | | F1 | CACTGAAGAATAAGTCAGTGACCCAGAAGACG |
|  |  | F2 | AGATGTGTATAAGAGACAGAGTCAGTGACCCAGAAGACGGAAGCA |
|  |  | R1 | TTTTCAAGTTTTTGGACTAAATTATCCCAACACC |
|  |  | R2 | AGATGTGTATAAGAGACAGTCCCAACACCGGGCAAAGTTATCC |
| c.2305G>T | | F1 | CCTTGTATTGAATTACTCATCTTTGCTCTCATGC |
|  |  | F2 | AGATGTGTATAAGAGACAGCTTTGCTCTCATGCTGCAGGCCATAG |
|  |  | R1 | CCTGAGCTGATCTGCTGGCATCTTGC |
|  |  | R2 | AGATGTGTATAAGAGACAGTCTGCTGGCATCTTGCAGTTTTCTGA |
| Exon 12 deletion | break 1 | F1 | TTCATAACAGAAATATTAAACAGGATAGTTCTCC |
|  |  | F2 | AGATGTGTATAAGAGACAGATTAAACAGGATAGTTCTCCATATTCTTCAGA |
|  | break 2 | R1 | TTTGTCCCTTTTATGATAAATGTCTATTTTGGTC |
|  |  | R2 | AGATGTGTATAAGAGACAGTGTCTATTTTGGTCTTTTGCCTATTTTTAATCAG |

Table S4. Primers used for Y chromosome in cfBEST.

| Site | Primer | Sequence |  |
| --- | --- | --- | --- |
| chrY site1 | F1 | TTTCGGCTTCAGTAAGCATTTTCCACTG |  |
|  | F2 | AGATGTGTATAAGAGACAGTCGGCTTCAGTAAGCATTTTCCACTGGTATCC |  |
|  | R1 | TCATCGTGTGGTCTCGCGATCAGA |  |
|  | R2 | AGATGTGTATAAGAGACAGCTCGCGATCAGAGGCGCAAGATGG |  |
| chrY site2 | F1 | AGCTTCTCCGGAGAGCGGGAATATTC |  |
|  | F2 | AGATGTGTATAAGAGACAGCTCCGGAGAGCGGGAATATTCTCTTG |  |
|  | R1 | GCACTCTCCTTGTTTTTGACAATGCAATCATATG |  |
|  | R2 | AGATGTGTATAAGAGACAGGACAATGCAATCATATGCTTCTGCTATGTTAAGC |  |
| chrY site3 | F1 | TGAGGTACGAATACAATACCCTAGCATCAATTCC |  |
|  | F2 | AGATGTGTATAAGAGACAGCGAATACAATACCCTAGCATCAATTCCACC |  |
|  | R1 | GCCAAGGCTGGGTGTTACCAGAAG |  |
|  | R2 | AGATGTGTATAAGAGACAGGGTGTTACCAGAAGGCAAAATCGTGC |  |

Table S5. The expected mutant ratio of the plasma of the pregnant woman.

| **Fetal gender** | **Maternal genotype** | **Fetal genotype** | **Expected mutant ratio** |
| --- | --- | --- | --- |
| female | N/N | N/N | 0% |
|  | N/N  (germline mosaicism） | N/M | FF/2 |
|  | N/M | N/N | 50%-FF/2 |
|  |  | N/M | 50% |
| male | N/N | N | 0% |
|  | N/N (germline mosaicism） | M | FF/(2-FF) |
|  | N/M | N | (1-FF)/(2-FF) |
|  |  | M | 1/(2-FF) |

M,mutant allele, N,wild-type allele,FF,fetal fraction
