# Supplementary material for: Noninvasive prenatal diagnosis of duchenne muscular dystrophy in five Chinese families based on relative mutation dosage approach
Source: BMC Med Genomics. 2021 Nov 22;14:275. doi: 10.1186/s12920-021-01128-1 (PMC8607717; doi:10.1186/s12920-021-01128-1)
Supplement: Supplementary file 2 — Additional file 2. Fig. S1. Schematic representation of the cfBEST method. Red dot: the site of interest; UMI: Unique Molecular identifiers; Index: sample index; Primer F1: Target-specific primer in 1st PCR; Primer F2: Target-specific primer in 2nd PCR, which is close to the site of interest; Primer U1: a universal primer of P7; Primer U2: a universal tail part of P5. [file 12920_2021_1128_MOESM2_ESM.pptx]

## Slide 1
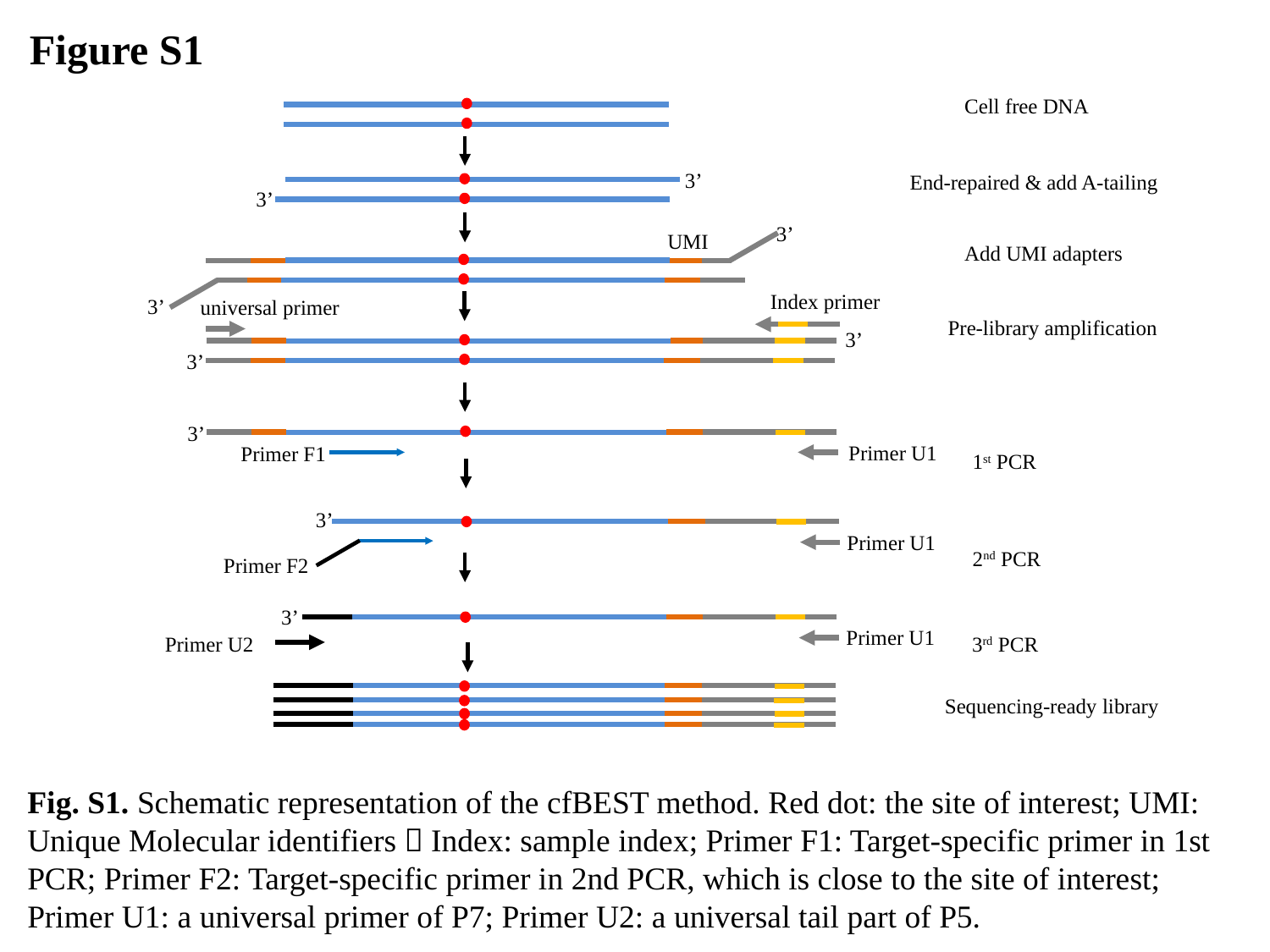

Figure S1
Cell free DNA
3’
End-repaired & add A-tailing
3’
3’
UMI
Add UMI adapters
Index primer
3’
universal primer
Pre-library amplification
3’
3’
3’
Primer U1
Primer F1
1st PCR
3’
Primer U1
2nd PCR
Primer F2
3’
Primer U1
Primer U2
 3rd PCR
 Sequencing-ready library
Fig. S1. Schematic representation of the cfBEST method. Red dot: the site of interest; UMI: Unique Molecular identifiers；Index: sample index; Primer F1: Target-specific primer in 1st PCR; Primer F2: Target-specific primer in 2nd PCR, which is close to the site of interest; Primer U1: a universal primer of P7; Primer U2: a universal tail part of P5.
