# Supplementary material for: Noninvasive prenatal diagnosis of duchenne muscular dystrophy in five Chinese families based on relative mutation dosage approach
Source: BMC Med Genomics. 2021 Nov 22;14:275. doi: 10.1186/s12920-021-01128-1 (PMC8607717; doi:10.1186/s12920-021-01128-1)
Supplement: Supplementary file 3 — Additional file 3. Fig. S2. Full-length gels of Fig. 3 are presented in Additional file 3: Fig. S2. [file 12920_2021_1128_MOESM3_ESM.pptx]

## Slide 1
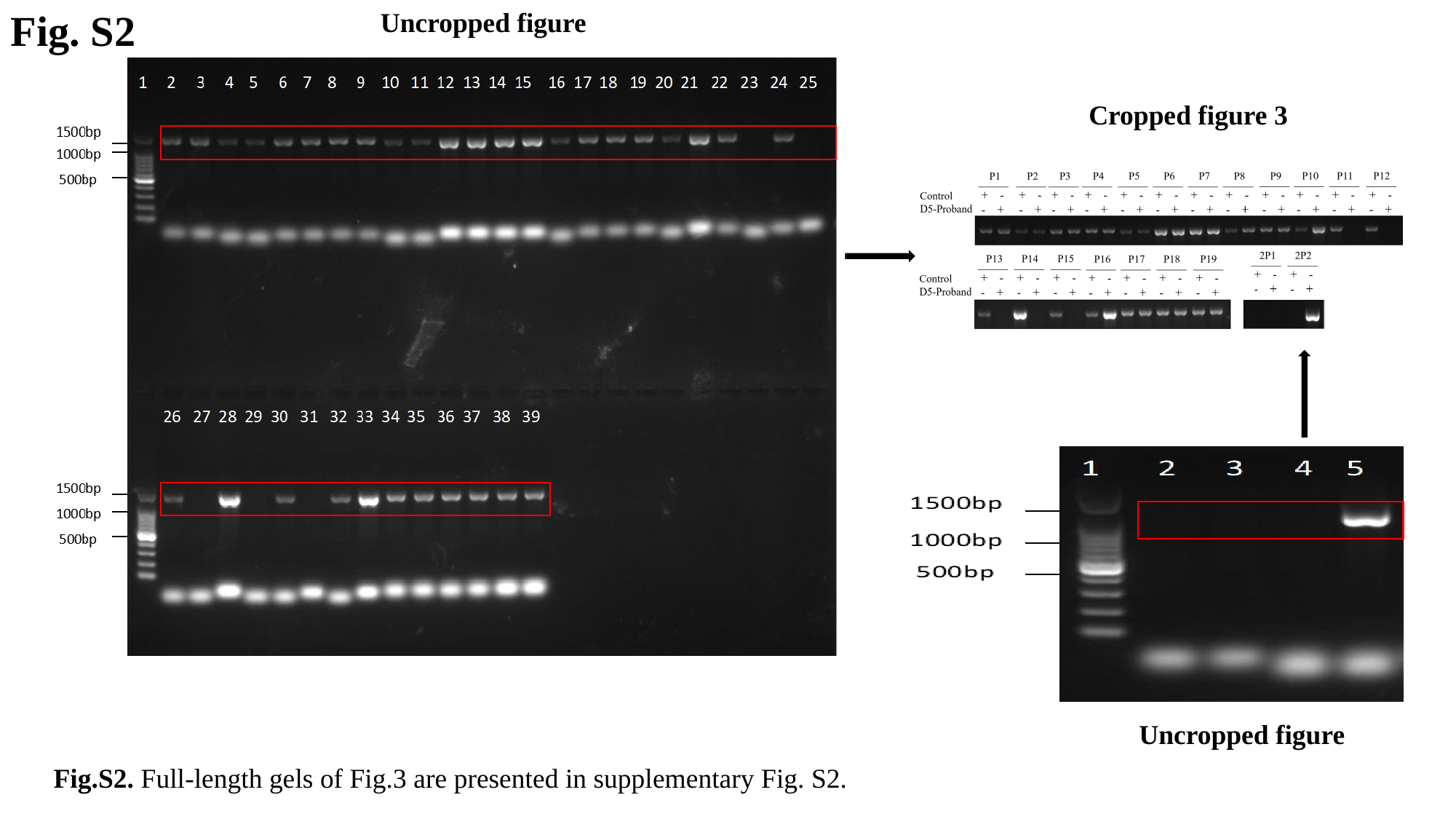

Uncropped figure
Cropped figure 3
Uncropped figure
Fig. S2
Fig.S2. Full-length gels of Fig.3 are presented in supplementary Fig. S2.
